# Influence of Oxidation on Electrical Properties of Compacted Cu Nanopowders

**DOI:** 10.1186/s11671-016-1700-6

**Published:** 2016-10-28

**Authors:** Volodymyr Nadutov, Anatoliy Perekos, Volodymyr Kokorin, Sergiy Konoplyuk, Taras Kabantsev

**Affiliations:** 1 G.V. Kurdyumov Institute for Metal Physics, National Academy of Science of Ukraine, Vernadsky Blvd. 36, Kyiv, 03680 Ukraine; 2 Institute of Magnetism, National Academy of Science of Ukraine and Ministry for Education and Science of Ukraine, Vernadsky Blvd. 36-b, Kyiv, 03680 Ukraine

**Keywords:** Electrical spark dispersion, Highly dispersed Cu nanopowders, X-ray diffraction analysis, Electric resistance

## Abstract

The phase composition and electrical transport properties of Cu powder obtained by electric spark dispersion and the pellets manufactured from this powder were studied by X-ray phase analysis and electric resistance measurements. The compacted powders were annealed in pure Ar atmosphere. It was shown that electrical resistance of the compacted Cu specimens essentially depends on the annealing temperature. In particular, the electrical resistance of the pellet after annealing at 873 K decreases on heating at low temperatures (semiconducting mechanism). As the temperature is increased, semiconducting behavior of resistivity is altered for metallic one. This change of conductivity type is ascribed to formation of metallic oxide and modification of its content during annealing.

## Background

The Cu-based Heusler alloys with restricted solubility are prospective materials for spintronic applications [1–3]. Nanopowders of the Cu-13.1 % Mn-12.6 % Al alloy were obtained by electrical spark dispersion (ESD) in different media including ethanol, gas, and water [4]. Electric conductivity of compacted powders (pellets) produced in ethanol and gas and annealed at 1073 K was found to be characteristic of semiconductors increasing with temperature in contrast to as-cast alloys, which demonstrate metallic-type conductivity.

Detailed analysis of all the results obtained led to conclusion that the type of conductivity is conditioned by formation of copper and manganese oxides in the process of preparation. The effect of copper oxide on electric conductivity is suggested to employ for controllable oxidization of pure copper to govern its conductivity. Thus, the aim of this study can be designated as determination of effect of phase content on electric conductivity of compacted copper powders prepared by successive procedures of electric spark dispersion, compacting, and annealing in argon atmosphere.

## Methods

The subjects of this investigation were highly dispersed powder (nanopowder) and compacted specimens of Cu. They were prepared from high purity Cu (99.99 %). Highly dispersed powder was produced by electrical spark dispersion in distilled water; specimens were made by pressing powder [5]. After ESD, powder particles were collected from operating fluid. First, they have been dried in air at room temperature and then at temperature of 200 °C for 1 h in drying oven to remove moisture. Next, powder has been compacted at room temperature, and finally, some of compacted specimens were annealed at 873 and 1073 K for 30 min in argon environment. X-ray diffraction measurements were carried out using diffractometer DRON −3.0 with cobalt anode. Diffraction patterns were analyzed by comparison of lines with maximum intensity corresponding to different crystalline phases. The size of coherently scattering domains (CSD) was calculated using the Scherrer equation [6].

## Results and Discussion

**Fig. 1:**
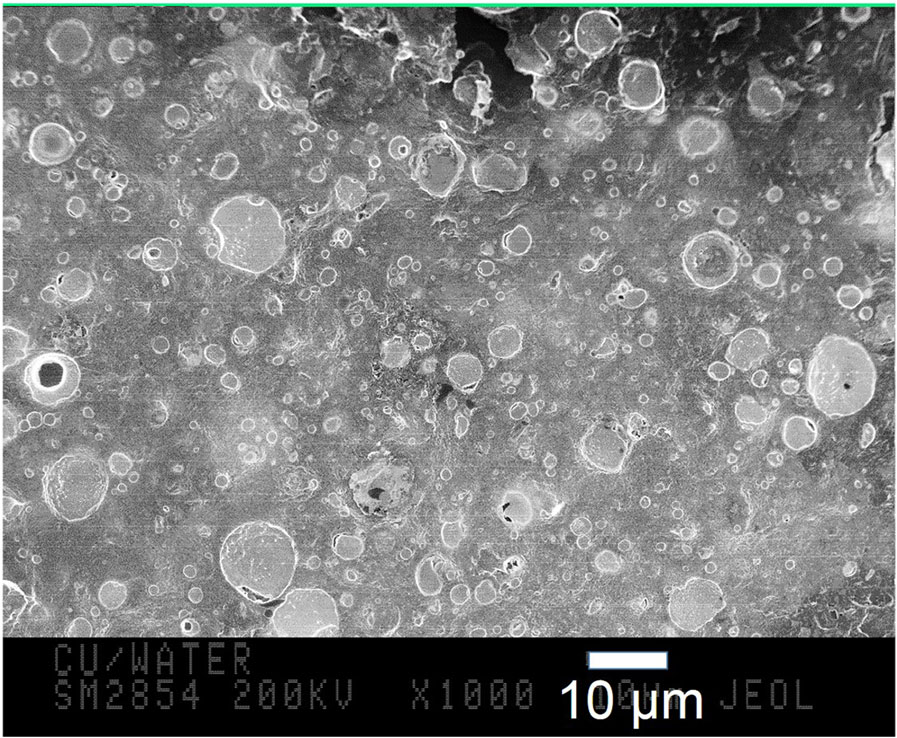
SEM micrographs of Cu powder particles produced by ESD in distilled water. Bright shell on their surface is oxide layer

The image of highly dispersed powder particles obtained by SEM is shown in Fig. 1. The particle-size distribution determined from this micrograph indicates that size (average diameter) of Cu particles ranges from 0.25 to 14 μm. Particles of 0.5 ÷ 7 μm are almost spherical. The part of the smallest particles having dimension smaller than 1 μm forms conglomerates. Furthermore, presence of globular particles of larger than 0.5 μm implies active agglomeration processes occurring most likely while drying. Notice that most of particles shown in Fig. 1 are coated with light-colored oxide shells, which is consistent with the X-ray analysis data concerning presence of oxides.

**Fig. 2:**
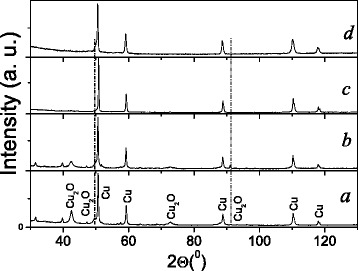
X-ray diffractograms of *a* Cu powder prepared by ESD in distilled water; *b* the pellets compacted from this powder without annealing and after annealing in Ar atmosphere for 30 min at 873 K (*c*) and 1073 K (*d*)

The results of X-ray diffraction for Cu powder after ESD in distilled water, for as-compacted specimen, and for the specimens annealed at temperatures of 873 and 1073 K in gaseous argon are shown in Fig. 2 and Table 1. These data indicate that the initial powder and the pellet after compacting contained a great deal of copper oxide Cu_2_O. Its amount was up to 38 % in the powder and 28 % in the pellet (Table 1). It is accounted for strong reaction between the Cu and the O atoms during ESD to form Cu_2_O. But annealing at 873 K led to decrease of Cu_2_O down to 2 % (Fig. 2c, Table 1). Furthermore, its lines disappear in the diffraction pattern after annealing at 1073 K (Fig. 2d, Table 1).

**Table 1:** Phase content and sizes of CSD (D) for as-received powder and pellets

Specimen	Constituent phases	Phase content, %	*D*, nm
Powder, after ESD	Cu	60	150
	Cu_2_O	40	15
Pellet, after compacting	Cu	70	170
	Cu_2_O	30	15
Pellet, after annealing at 873 K for 30 min	Cu	98	>500
	Cu_2_O	2	130
Pellet, after annealing at 1073 K for 30 min	Cu	100	>500
	Cu_2_O	–	

**Fig. 3:**
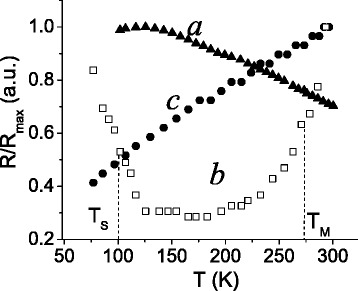
Electric resistance of *a* as-compacted Cu pellet; Cu pellets annealed at 873 K (*b*) and at 1073 K (*c*) vs. temperature. See the main text for details

The change of electric resistance *R* with temperature for the pellets listed in Table 1 is illustrated in Fig. 3. The temperature dependence of the as-compacted pellet was found to demonstrate semiconductor behavior similar to the Cu-13.1 % Mn-12.6 % Al alloy [4]. As is seen, its resistance decreases at temperatures above 120 K (Fig. 3a). By contrast, electric resistance of the pellet annealed at 1073 K clearly shows an opposite trend increasing with the temperature in the whole temperature interval of measurements. This is intrinsic to materials with metallic-type conductivity (Fig. 3c). Electric resistance of the pellet subjected to annealing at 873 K exhibits the behavior reflecting intermediate character of conductivity. Decreasing *R* within the temperature interval from 77 to 120 K is changed for its increasing above 180 K (Fig. 3b).

The variation of electric resistance for the compacted powders can be explained from results of X-ray phase analysis (Fig. 2, Table 1). It is evident that semiconducting character of conductivity is caused by the formation of copper oxide during ESD. Its content reduces due to decomposition as temperature of annealing is increased. These results are consistent with those [4] where semiconducting behavior associated with presence of copper and manganese oxides was reported for compacted specimens of highly dispersed Cu-13.1 % Mn-12.6 % Al powder. It should be noted that in case of disordered intermetallic alloys, semiconducting behavior can be attributed to strong atomic scattering, which results in quantum interference effects such as a weak localization or hopping conductivity [7–10]. Nevertheless, analysis of all collected experimental results allowed authors to conclude that the Cu-13.1 % Mn-12.6 % Al-compacted powders comprised of ordered and disordered phases demonstrated semiconducting behavior owing to appearance of oxide layer on the surface of particles rather than by other reasons [4]. Here, the compacted Cu powder specimens contain only copper and copper oxides without any disordered phase constituents. This and the other experimental data unambiguously confirm correlation between degree of oxidization of highly dispersed Cu particles and conduction mechanism.

As it follows from the X-ray diffraction data, thickness of oxide layers on the surface of Cu particles forming pellets is reduced with decreasing the content of Cu oxide in specimen and hence in particles. That is why, conductivity type depends on temperature of annealing. This contention is also supported by resistivity behavior at low-temperature characteristic of semiconductors in spite of only 2 % of Cu_2_O in the specimen annealed at 873 K (Fig. 3b). If nanoparticles of Cu_2_O had been distributed in the pellet regardless of Cu nanoparticles, resistivity of Cu_2_O phase would never have effect on total resistivity being bridged by several orders lower resistivity of Cu. In fact, the measurements of *R* (Fig. 3b) show that below 120 K, semiconducting copper oxide phase dominates temperature behavior of resistivity and even at higher temperatures up to 180 K, there are no signs of prevalence of metallic type of conductivity associated with Cu phase. It is possible only if Cu_2_O phase develops mainly on the surface of highly dispersed particles of Cu.

It is well known that resistivity of semiconductors exponentially decreases with the temperature due to enhancing the thermal activation, which excites electrons to the conduction band. In contrast, the resistivity of metals increases with the temperature following linear dependence at all temperatures except for low ones. The mixture of phases with different types of conductivity present in the alloy annealed at 873 K shows more complicated change of resistivity with the temperature. Switching observed between semiconductor and metallic types of conductivity with the temperature (Fig. 3b) can be confirmed using Lichtenecker’s formula for resistivity of two-phase alloys [11]:

1 $$ \rho (T)={\rho}_{{\mathrm{Cu}}_2\mathrm{O}}^C(T){\rho}_{\mathrm{Cu}}^{1-C}(T), $$

where ${\rho}_{{\mathrm{Cu}}_2\mathrm{O}}$ and *ρ*_Cu_ are the resistivities of copper oxide and pristine Cu, respectively, and *c* is the volume fraction of Cu_2_O phase.

Without going into details of temperature dependence of resistivity over the whole temperature interval of the measurements, one can restrict the consideration to temperatures *T*_S_ = 100 K and *T*_M_ = 273 K, where respective semiconducting and metallic behavior of resistivity are well defined.

Let us estimate the ratio of $$ \raisebox{1ex}{$\rho (T)$}\!\left/ \!\raisebox{-1ex}{$\rho \left(T+\varDelta T\right)$}\right. $$ (2) at *T*_S_ and *T*_M_ while ∆*T* = 1 K. The value of this ratio will indicate the type of conductive mechanism at particular temperatures. Substitution of the expressions $$ {\rho}_{{\mathrm{CuO}}_2}(T)=A \exp \left(\frac{E_g}{2kT}\right) $$ and *ρ*_Cu_(*T* + *ΔT*) = *ρ*_Cu_(*T*)(1 + *αΔT*) where *A* is a constant, *E*_*g*_ is the energy gap in Cu_2_O, *α* is the temperature coefficient of Cu resistivity into (1) and (2) yields

$$ \raisebox{1ex}{$\rho (T)$}\!\left/ \!\raisebox{-1ex}{$\rho \left(T+\varDelta T\right)$}\right.=\raisebox{1ex}{${\rho}_{{\mathrm{Cu}\mathrm{O}}_2}^C(T){\rho}_{\mathrm{Cu}}^{1-C}(T)$}\!\left/ \!\raisebox{-1ex}{${\rho}_{{\mathrm{Cu}\mathrm{O}}_2}^C\left(T+\varDelta T\right){\rho}_{\mathrm{Cu}}^{1-C}\left(T+\varDelta T\right)$}\right.\approx \raisebox{1ex}{$ \exp \left(\frac{c{E}_g\varDelta T}{2k{T}^2}\right)$}\!\left/ \!\raisebox{-1ex}{${\left(1+\alpha \varDelta T\right)}^{1-c}$}\right. $$

Taking into account that *E*_*g*_ = 2 eV [12], *c* = 0.02, *α* = 0.004, finally, we have

$$ \raisebox{1ex}{$\rho \left({T}_S\right)$}\!\left/ \!\raisebox{-1ex}{$\rho \left({T}_S+\varDelta T\right)$}\right.\approx 1.02>1\kern1.5em \mathrm{and}\kern1em \raisebox{1ex}{$\rho \left({T}_M\right)$}\!\left/ \!\raisebox{-1ex}{$\rho \left({T}_M+\varDelta T\right)$}\right.\approx 0.99<1 $$

This result suggests that at *T*_*S*_, resistivity decreases while at *T*_*M*_, it increases with the temperature in accordance with the experimental data (Fig. 2b).

## Conclusions

Electric conductivity of compacted Cu powders prepared by ESD method and annealed in Ar environment varies with the annealing temperature as oxidation level of powder particles is changed. Appearance of semiconducting Cu_2_O phase modifies metallic-type behavior of electric conductivity causing its growth with the temperature.

## References

[1] Konoplyuk SM, Kokorin VV, Kolomiets OV, Perekos AE, Nadutov VM (2011). Magnetoresistance of Cu-Mn-Al melt-spun ribbons containing the system of interacting ferromagnetic inclusions. JMMM.

[2] Konoplyuk SM, Kozlova LE, Kokorin VV, Perekos AE, Kolomiets OV (2016). Magnetic states in ensemble of ferromagnetic nanoparticles in Cu-Mn-Al alloy. Nanoscale Res Lett.

[3] Kozlova LE, Bondarenko VO, Kokorin VV, Konoplyuk SM (2015). Variation of Seebeck coefficient at martensitic transformation in Cu–Mn–Al alloy. Mater Lett.

[4] Nadutov VM, Perekos AE, Kokorin VV, Konoplyuk SM, Zalutskiy VP, Efimova TV (2014). Effect of electric-spark dispergation on magnetic and electrical-transport properties of Heusler Cu-Mn-Al alloy. Metallofizika i Noveishie Tekhnologii.

[5] Nadutov VM, Perekos AE, Kokorin VV, Konoplyuk SM, Hranovska KM (2015). Method of fabrication of nanostructural thermoelectric semiconductor alloy.

[6] Holzwarth U, Gibson N (2011). The Scherrer equation versus the ‘Debye-Scherrer equation’. Nat Nanotechnol.

[7] Anderson PW, Abrahams E, Ramakrishan TV (1979). Possible explanation of nonlinear conductivity in thin-film metal wires. Phys Rev Lett.

[8] Jazbec S, Koželj P, Vrtnik S, Jagličić Z, Popčević P, Ivkov J, Dolinšek J (2012). Electrical, magnetic, and thermal properties of the δ-FeZn_10_ complex intermetallic phase. Phys Rev B.

[9] Yu D, Wang C, Wehrenberg BL, Guyot-Sionnest P (2004). Variable range hopping conduction in semiconductor nanocrystal solids. Phys Rev Lett.

[10] Kudryavtsev YV, Perekos AO, Uvarov NV, Kolchiba MR, Synoradzki K, Dubowik J (2016). Mixed structural face-centered cubic and body-centered cubic orders in near stoichiometric Fe_2_MnGa alloys. J Appl Phys.

[11] Lifshits BG, Kraposhin VS, Linetskiy YL (1980). Physical properties of metals and alloys.

[12] Meyer BK, Polity A, Reppin D, Becker M, Hering P, Klar PJ, Sander T, Reindl C, Benz J, Eickhoff M, Heiliger C, Heinemann M, Bl¨asing J, Krost A, Shokovets S, M¨uller C, Ronning C (2012). Binary copper oxide semiconductors: from materials towards devices. Phys Status Solidi B.

